# Activation of the noncanonical inflammasome-GSDMD pathway triggers pyroptosis in bone marrow and promotes periosteal bone formation

**DOI:** 10.1093/jbmr/zjaf197

**Published:** 2025-12-18

**Authors:** Wei Zou, Chun Wang, Yongjia Li, Wentong Jia, Steven L Teitelbaum, Gabriel Mbalaviele

**Affiliations:** Division of Bone and Mineral Diseases, Washington University School of Medicine, St. Louis, MO 63110, United States; Division of Anatomic and Molecular Pathology, Department of Pathology and Immunology, Washington University School of Medicine, St. Louis, MO 63110, United States; Division of Bone and Mineral Diseases, Washington University School of Medicine, St. Louis, MO 63110, United States; Division of Bone and Mineral Diseases, Washington University School of Medicine, St. Louis, MO 63110, United States; Division of Laboratory and Genomic Medicine, Washington University School of Medicine, St. Louis, MO 63110, United States; Division of Bone and Mineral Diseases, Washington University School of Medicine, St. Louis, MO 63110, United States; Division of Anatomic and Molecular Pathology, Department of Pathology and Immunology, Washington University School of Medicine, St. Louis, MO 63110, United States; Division of Bone and Mineral Diseases, Washington University School of Medicine, St. Louis, MO 63110, United States

**Keywords:** Inflammasome, gasdermin, pyroptosis, inflammation, osteoprogenitors, bone formation

## Abstract

Evidence indicating that inflammation is commonly associated with ectopic osteogenesis in certain autoimmune and infectious conditions challenges the dogma that inflammatory responses always suppress bone formation. In this study, we find that systemic administration of lipopolysaccharide (LPS) to mice causes not only inflammation in bone marrow, as expected, but also stimulates periosteal bone formation. This response can be reproduced in vitro as bone marrow supernatants from LPS-treated mice induce robust osteogenesis of osteoprogenitors compared to supernatants from PBS-treated counterparts. Periosteal bone accrual is partly dependent on periosteal leptin receptor-positive (LepR)^+^ osteoprogenitors but not bone marrow LepR^+^ or adiponectin (Adq)^+^ osteoprogenitors and correlates with pyroptosis within bone marrow. Consistent with the dependence of periosteal osteogenesis on pyroptosis, this response is slightly attenuated in *Nlrp3^−/−^* or *caspase-1^−/−^* mice but significantly inhibited in *caspase-11^−/−^*, *caspase-1^−/−^*;*caspase-11^−/−^*, or *Gsdmd^−/−^* mice. Our study reveals a novel role for pyroptosis in which lysed cells release intracellular contents that stimulate osteoprogenitors and promote osteogenic differentiation within the periosteal compartment.

## Introduction

Bone mass is decreased in many diseases, including osteoporosis, inflammatory arthritis, and malignancies, and is associated with increased risks for the development of late fractures, and morbidities in the elderly populations.^1^^,^^2^ In fact, it was reported that 20%-50% of geriatric patients (≥65 yr) with a hip fracture die within 1 yr of fracture.^3^ Although significant progress has been made in the treatment of low bone mass disorders, there remains an unmet medical need for the development of therapeutic agents that specifically improve the strength of cortical bone, which comprises most of skeletal bone mass (80%) at sites, such as the hip and wrist, and provides approximately 65%-90% of proximal femur strength.^4^^,^^5^ A better understanding of nuances between these processes will lead to the development of safer and effective therapeutics for the treatment of disorders, such as osteoporosis and delayed fracture healing.

Bone mass and quality are maintained throughout the lifespan by a tightly balanced process mediated by bone-resorbing osteoclasts (OCs) and bone-forming osteoblasts (OBs).^6^ While OCs arise from hematopoietic stem cells, OBs originate from mesenchymal stem cells.^7–12^ Within adult bone marrow, leptin receptor-expressing (LepR) osteoprogenitors are the major source of new OBs for bone maintenance.^10^ These cells or their subsets also express adiponectin (Adq), Cxcl12, or Ebf3.^8^^,^^9^^,^^11–13^ An imbalance whereby bone resorption outpaces bone formation underlies the pathogenesis of diseases of bone loss.^14^ The current dogma posits that inflammation tilts this balance in favor of bone resorption and decreased bone formation.^7^^,^^15^ This view is supported by clinical data indicating that high levels of inflammatory factors, such as IL-6 and TNF, are associated with a higher risk of bone fracture, and overwhelming preclinical evidence indicating that these cytokines stimulate bone resorption while inhibiting bone formation.^16^^,^^17^ Contrasting these findings are numerous studies showing that inflammation can promote osteogenesis. Indeed, several human infectious and non-infectious inflammatory conditions, such as ankylosing spondylitis,^18^^,^^19^ osteomyelitis,^20^ and fracture healing^21^ are associated with cortical bone accrual. In support of this clinical evidence, experimental osteomyelitis in which *Staphylococcus aureus* is injected into femoral diaphysis is characterized by extensive cortical bone loss associated with robust reactive cortical bone formation surrounding the site of infection.^22^^,^^23^ Similarly, administration of TLR2 or TLR4 agonists into mouse calvaria induces bone formation through canonical Wnt signaling pathways.^24^ In sum, although inflammation promotes bone resorption, it can give rise to bone formation, an outcome that is probably tissue context dependent. Coincidentally, inflammation predominantly induces reactive periosteal bone formation.^19^^,^^22^^,^^23^^,^^25^

Emerging research implicates lytic forms of programed cell death, including pyroptosis, necroptosis, and PANoptosis, in the propagation of inflammation.^26^^,^^27^ Pyroptosis is caused by cell stressors, such as lipopolysaccharide (LPS), a component of the outer membrane of gram-negative bacteria and is mediated by a family of proteins called gasdermins (GSDMs) of which GSDMD is the most studied family member.^28–30^ Cleavage of GSDMD by caspase-1, caspase-11, or neutrophil elastase generates GSDMD amino-terminal fragments, which form plasma membrane pores.^28–30^ GSDMD conduits facilitate the secretion of IL-1β and IL-18 by live cells, but excessive pore formation can compromise the integrity of the plasma membrane and causes pyroptosis, leading to the release of not only IL-1β and IL-18, but also intracellular contents.^29^^,^^30^ Thus, GSDMD pores can exacerbate inflammation through pyroptosis-dependent mechanisms. We and others have previously reported that inflammasome-GSDMD pathways play an important role in bone development and maintenance in humans and rodents.^31–33^ Consistent with its functional link to inflammasomes, LPS stimulates bone resorption by upregulating OC differentiation and activity in disease models.^34^ LPS is found in systemic circulation in patients with increased gut permeability or decreased liver functions, and has been linked to multiple conditions, including skeletal diseases.^35–37^ Thus, LPS, inflammasomes, and GSDMD are involved in bone metabolism. However, the extent to which pyroptosis per se, impacts bone pathophysiology is unknown.

In this study, we have identified a novel mechanism in which LPS activates the noncanonical inflammasome pathway, leading to GSDMD-mediated pyroptosis in bone marrow cells. This response ultimately induces periosteal cells, including LepR^+^ cells to differentiate into OBs and form bone.

## Materials and methods

### Mice and reagents

*Rosa-DtA* (*Lox-Stop-Lox-ROSA-DTA*) mice, Col1a1^*^2.3GFP mice, *Adipoq-Cre* mice, *Lepr-Cre* mice, *LysM-Cre* mice, *tdTomato* (Ai9) mice, *Nlrp3^−/−^* mice, *Il1r^−/−^* mice, *Casp1^−/−^* mice, *Casp11^−/−^* mice, *Casp1^−/−^;11^−/−^* mice, and *Gsdmd^−/−^* mice were purchased from The Jackson Laboratory. Herpes simplex virus *thymidine kinase* (*TK*)*-3.6Col1a1* (Tk^Col1^*^3.6^) mice^38^ were kindly provided by Robert Jilka and Charles O’Brien (University of Arkansas). All animals were housed in the specific pathogen free animal facility of Washington University School of Medicine, where they were maintained at 22 °C in a 12-h light–dark cycle. Animal work was performed according to the policies of IACUC at Washington University School of Medicine in St. Louis. Mice were analyzed under approved IACUC protocols.

Mice were crossed in-house to generate Adipoq^Cre^; Col1^*^2.3GFP; Ai9 (*tdT^Adq^*; GFP^Col^*^2.3^) and *Lepr^Cre^*;Col1^*^2.3GFP;Ai9 (*tdT^LepR^*;GFP^Col^*^2.3^) on a C57Bl/6 background. Ganciclovir (Acros Organics, Cat# 461710050) was injected at 8 μg/g i.p. twice daily in saline to ablate TK^+^ cells. LPS from *E. coli* (Sigma-Aldrich, L2630) or *Salmonella* (Sigma-Aldrich, L6511) was given at 1 mg/kg i.p. as indicated. LPS, collagenase I, and collagenase IV were purchased from Sigma.

### Micro-computed tomography

The whole femur was scanned using μCT50 scanner (Scanco Medical AG; 70 kVp, 57 μA, 700 ms integration time, and 7.4 μm voxel size). A lower threshold of 450 mg HA/ccm was used for evaluation of trabecular bone. For cortical bone, lower thresholds of 580 and 800 mg HA/ccm were used to assess total cortical bone and existing (consolidated) cortical bone, respectively. Fifty slices were analyzed for both midshaft and metaphyseal cortical bone.

### Biomechanical testing

Three-month-old WT male mice were i.p. injected with PBS or 1 mg/kg LPS from *E. coli* on days 0, 4, and 8. Mice were sacrificed on day 28. Femur biomechanical properties were assessed using a 3-point bending test as previously described.^39^ Briefly, femurs were dissected and cleaned of all soft tissue. Femora were scanned by micro-computed tomography (μCT) at the midshaft using Scanco μCT50 scanner to determine cross-sectional geometric properties. They were then mechanically tested to failure in 3-point pending (Instron 8841; support span: 7 mm; displacement rate: 0.1 mm/s). Failure occurred directly beneath the loading point, at the 50% length of the femur. Force-displacement data were collected and analyzed to determine whole-bone (structural) mechanical properties (stiffness (N/mm), yield force (N), maximum (N), and work-to-fracture (N^*^mm)).

### Histology and histomorphometry

Femurs and tibias were fixed in 10% neutral buffered formalin, followed by decalcification in 14% EDTA for 10 d, paraffin embedding, and TRAP or H&E staining.

### Microscopic analysis

Bones were fixed with 10% neutral buffered formalin 24-48 h at room temperature and decalcified in 14% EDTA for 3 d. The bones were then infiltrated with 30% sucrose overnight at 4 °C. About 10-μm-thick cryoprotection sections were mounted with antifade mounting medium with DAPI (Vector Laboratories), and images were acquired with a confocal microscope (Nikon C-1 confocal system).

### TUNEL staining

ApopTag Peroxidase in Situ Apoptosis Detection Kit was purchased from Sigma-Aldrich. Paraffin sections (4-μm-thick) were subjected to TUNEL staining according to the manufacturer’s instructions and analyzed using ImageJ. The percentage of TUNEL^+^ cells was determined by dividing the number of TUNEL^+^ cells by the total number of cells.

### Reporter bone analysis

Femurs of tdT^lepR^;GFP^Col1^*^2.3^ or tdT^Adq^;GFP^Col1^*^2.3^ were fixed in 10% neutral buffered formalin, followed by decalcification in 14% EDTA for 3 d. The bones were then infiltrated with 30% sucrose overnight at 4 °C for cryoprotection and embedded in optimal cutting temperature (Tissue-Tek). Sections of 10-μm thickness were prepared with a Leica cryostat equipped with Cryojane (Leica). tdT^+^ and GFP^+^ cells were analyzed using ImageJ.

### Bone marrow supernatant and blood collection and ELISA analysis

After removing the epiphysis, the tibias and femurs were centrifuged at 900 × g in a tube containing PBS (100 μL/mouse) and supernatants were kept at −80 °C. Blood was drawn from submandibular vein with BD Microtainer collection tubes. Cell death was assessed by detection of lactate dehydrogenase (LDH) in marrow supernatant and serum using the LDH Cytotoxicity Detection Kit (Takara). IL-1β levels in marrow supernatant and serum were measured by an ELISA kit (eBioscience).

### Proteomics analysis

Mouse bone marrow was centrifuged in a tube containing 100 μL of PBS. Collected samples were used for proteomics analysis by the Mass Spectrometry Technology Access Center at McDonnell Genome Institute (MTAC@MGI) at Washington University School of Medicine. Briefly, after measuring protein concentrations, each sample was reduced, alkylated, and digested with trypsin according to the optimized MTAC’s SOP. Digested peptides were desalted on C18 spin columns, quantified and the same normalized amount of peptides were analyzed by LC–MS/MS using an Orbitrap instrument online with nanoUHPLC. Data were searched against a mouse database using MaxQuant search engine and subjected to label-free quantification (LFQ) based on the MS1 peptide intensity. Proteins were filtered for >1 unique peptide. For statistical analysis, the LFQ intensities will be log2 transformed and grouped into biological groups. Imputation was applied to fill in missing values based on normal distribution, which is recommended for LFQ. Pair-wise *t*-test with FDR correction was employed to determine significant differences among the groups.

### Western blot analysis

Three-month-old WT and *Gsdmd^−/−^* mice were i.p. injected with PBS or LPS on day 0 and sacrificed on day 3. The supernatants from centrifuged bone marrow were analyzed by immunoblotting. Protein concentrations were determined by the Bio-Rad Laboratories method, and equal amounts of proteins were subjected to SDS-polyacrylamide gel electrophoresis using 12% or 15% gels. Proteins were transferred onto nitrocellulose membranes and incubated with antibodies against Lamp2 (DSHB, UIowa), Creld2 (DSHB, UIowa), S100a6 (DSHB, UIowa), and CALR-1(DSHB, UIowa) overnight at 4 °C followed by incubation at room temperature for 1 h with secondary goat anti-mouse IRDye 800 (Thermo Fisher Scientific), goat anti-rat Alexa Fluor 680 (Molecular Probes) or goat anti-rabbit Alexa Fluor 680 (Thermo Fisher Scientific), respectively. The results were visualized using the Odyssey Infrared Imaging System (LI-COR Biosciences).

### Isolation of bone marrow cells and periosteal cells and osteoblast differentiation

Bone marrow cells and periosteal cells were isolated by collagenases digestion as previously described.^40^^,^^41^ After 1 wk expansion in αMEM with 20% FBS, 5 × 10^4^ cells/well/mL were seeded into 24-well plate for 3 d after which osteoblast differentiation medium containing 50 μg/mL ascorbic acid and 2 mM β-glycerophosphate in αMEM was added. Cells were stained for alkaline phosphatase (ALP) activity.

### Flow cytometry analysis

After removing the epiphysis, the tibias and femurs were centrifuged to collect the bone marrow. Cells were washed with sterile DPBS and pelleted by centrifugation at 500 × *g* for 5 min at 4 °C. The 2× primary antibody cocktail is comprised of rat anti-mouse SiglecF-BV421 (clone E50-2440, BD Horizon, 562681), rat anti-mouse/human CD11b-Pacific Blue (clone M1/70, BioLegend, 101224), rat anti-mouse Ly6C-BV570 (clone HK1.4, BioLegend, 128030), rat anti-mouse F4/80-BV650 (clone BM8, BioLegend, 123149), rat anti-mouse Ly6G-BV711 (clone 1A8, BioLegend, 127643), Amenian hamster anti-mouse TCRβ-Alexa Fluor 488 (clone H57-597, BioLegend, 109215), rat anti-mouse CD45-PerCP (clone 30-F11, BioLegend, 103130), mouse anti-mouse CD64-PE/Dazzle594 (clone X54-5/7.1, BioLegend, 139320), Armenian hamster anti-mouse CD11c-PE/Cy5.5 (clone N418, Invitrogen/eBioscience, 35-0114-82), rat anti-mouse MHC-II-PE/Fire 810 (clone M5/114.15.2, BioLegend, 107667), and mouse anti-mouse NK1.1-APC/Fire750 (clone PK136, BioLegend, 108752). Following a 30-min incubation on ice, cells were washed twice with FACS buffer and resuspended in 200 μL FACS buffer. An aliquot of 100 μL was immediately analyzed live on a Cytek Aurora spectral flow cytometer.

### Data representation and statistical analysis

Data are expressed as mean ± SD. Statistical significance was determined using unpaired Student’s 2-tailed *t*-test, one-way ANOVA test or chi-square test. ^*^p < .05, ^**^p < .01, ^***^p < .001 in all experiments.

## Results

### LPS stimulates not only bone resorption, but also periosteal bone formation

**Figure 1:**
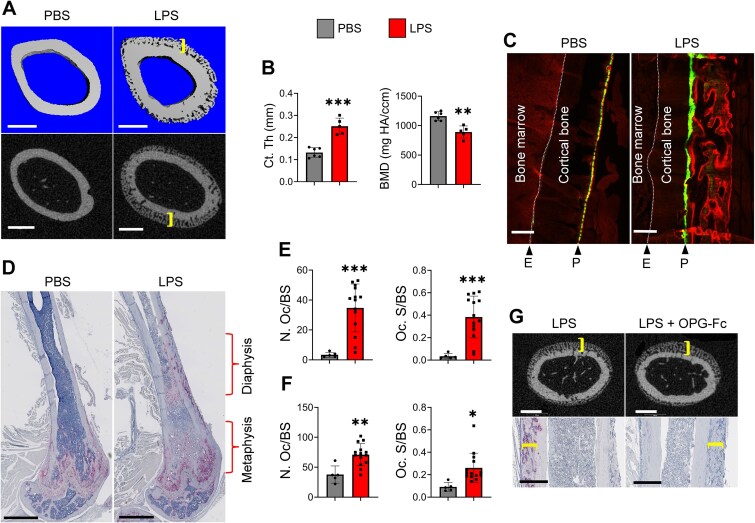
LPS stimulates periosteal bone formation, a response that is decoupled from bone resorption. (A-F) Three-month-old female WT mice (A and B) or male mice (C-G) were i.p. injected with PBS or 1 mg/kg LPS from *E. coli* on days 0, 4, and 8, mice were sacrificed on day 10. (A) Representative μCT images of femoral diaphysis treated with PBS (*n* = 6) and LPS (*n* = 5). Scale bar: 500 μm. (B) Cortical thickness (Ct.Th) and BMD analysis of (A). (C) WT mice were i.p. injected with PBS (*n* = 5) or LPS (*n* = 11) on days 0, 4, and 8, then with calcein and alizarin red on days 4 and 8, respectively. Representative images of calcein- and alizarin red-labeled femurs. Scale bar: 200 μm. E, endosteum; P, periosteum. (D-F) Femoral sections were stained for TRAP activity. (D) Representative images. Scale bar: 1 mm. (E) Quantitative analysis of diaphyseal areas. (F) Quantitative analysis of metaphyseal areas. N.OC/BS, OC number/bone surface. OC.S/BS, OC surface/bone surface. (G) Three-month-old WT mice were i.p. injected with 1 mg/kg LPS with (*n* = 10) or without OPG-fc (5 mg/kg/mouse; *n* = 10) on days 0, 4, and 8. Mice were sacrificed on day 10 and femurs were collected for μCT analysis (top; scale bar: 500 μm) and TRAP staining (bottom; scale bar: 400 μm). Representative images of femoral diaphysis. Brackets indicate the thickness of the newly formed woven bone, which was histologically identified by its characteristic porous appearance. ^*^p < .05, ^**^p < .01, ^***^p < .001. Unpaired *t*-test.

We previously generated *Dtr^Adq^* mice bearing primate diphtheria toxin receptor (Dtr) downstream of a floxed-stop codon and expressing *Adq-Cre*. We found that elimination of Adq^+^ cells upon injection of diphtheria toxin (DT) caused systemic increased of bone mass.^39^ To determine the role that inflammation plays in this response, we exposed DT-treated *Dtr^Adq^* mice to a low dose of *E. coli*-derived LPS for 10 d. While LPS administration decreased bone formation in bone marrow cavity of Adq^+^ cell-ablated mice, it dramatically and uniquely promotes periosteal bone formation (Figure S1A). To rule out the influence of DT in this response, we subjected WT mice to a similar LPS regimen. While LPS had no effect on the endosteal bone envelope, it induced massive periosteal bone formation (Figure 1A and B). While LPS significantly enhanced cortical bone parameters, including thickness and area as well as periosteal perimeter, it did not affect bone marrow area, endocortical perimeter, and trabecular bone (Figure 1A and B and Figure S1B and C). Periosteal osteogenesis also occurred in the tibia, humerus, and ilium (Figure S2). To ensure that induction of periosteal osteogenesis was not limited to *E. coli*-derived LPS, mice were also side-by-side inoculated with an identical dose of LPS (1 mg/kg) from *E. coli* or *Salmonella enterica*. *Salmonella*’s LPS increased cortical bone thickness without affecting trabecular bone mass in a similar manner to *E. coli*’s LPS (Figure S3A and B). The similarity of bone alterations induced by LPS from two different bacterial strains suggest that these responses are driven by LPS-intrinsic properties.

**Figure 2:**
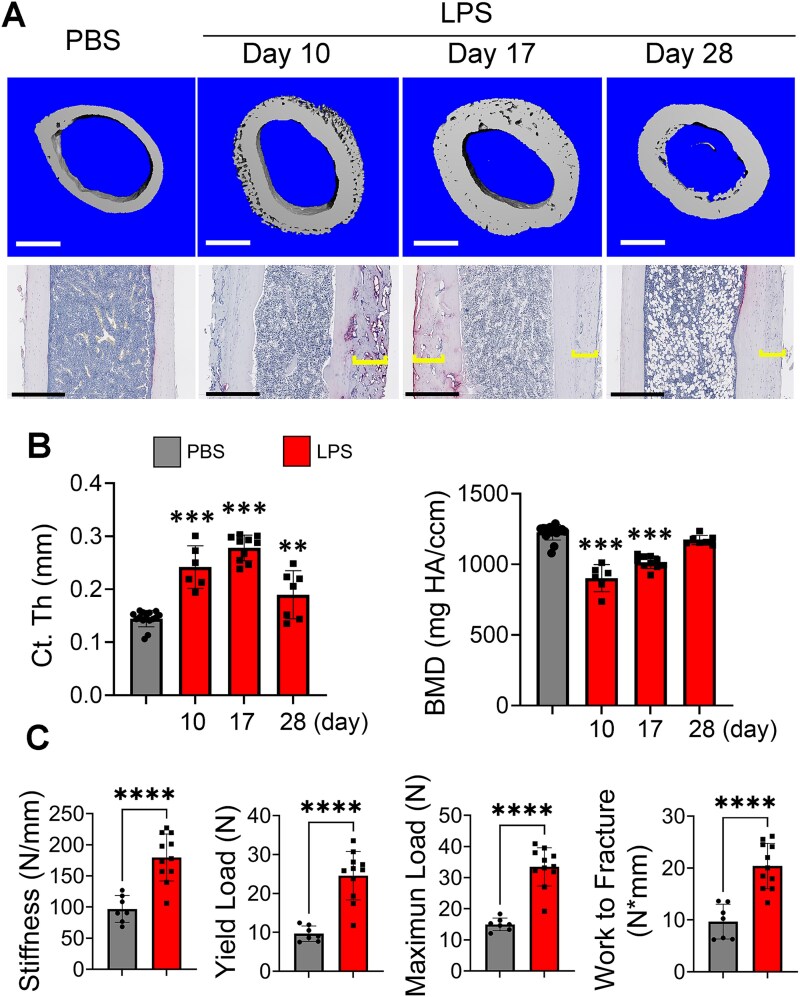
LPS-induced periosteal woven bone undergoes remodeling into mature bone. Three-month-old female WT mice were i.p. injected with PBS (*n* = 15) or 1 mg/kg LPS from *E. coli* (*n* = 6-10) on days 0, 4, and 8. Mice were sacrificed on days 10, 17, or 28. (A) Upper panel: representative μCT images of femoral diaphysis are shown. Scale bar: 500 μm. Lower panel: representative femoral sections stained for TRAP activity. Brackets indicate the thickness of the newly formed bone. Scale bar: 400 μm. (B) Cortical thickness (Ct.Th) and BMD analysis at femur midshaft of (A). (C) Three-month-old WT male mice were i.p. injected with PBS or 1 mg/kg LPS from *E. coli* on days 0, 4, and 8. Mice were sacrificed on day 28. Femur biomechanical properties were assessed using a 3-point bending test. ^**^p < .01; ^***^p < .001; ^****^p < .0001. (B) One-way ANOVA test. Unpaired t-test

Focusing on *E. coli*-derived LPS, calcein and alizarin red double labeling experiments confirmed accrual periosteal but not endosteal bone formation in response to LPS treatment (Figure 1C). We also unexpectedly found that LPS decreased cortical BMD (Figure 1B), presumably due to increased OC-mediated bone resorption. Consistent with this view, although LPS treatment did not alter the cortical thickness of the existing bone, it nevertheless reduced its BMD (Figure S4A-D) and enhanced OC number and surface not only in metaphyseal, but also in diaphyseal bone regions (Figure 1D-F). Thus, LPS-induced bone resorption throughout the femur, but reactive bone formation occurred mainly in the diaphyseal area. To determine whether periosteal bone formation and resorption induced by LPS were coupled, we injected WT mice with LPS in the presence or absence of osteoprotegerin-Fc (OPG-Fc) to block OC differentiation. While administration of OPG-Fc completely inhibited OC formation in both metaphyseal and diaphyseal bone regions, it did not affect periosteal osteogenesis (Figure 1G and Figure S5). To determine whether the periosteal woven bone undergoes remodeling into mature bone, both PBS- and LPS-exposed samples were analyzed not only on day 10, but also on days 17 and 28. Periosteal bone thickness increased in the diaphysis (Figure 2A and B) and metaphysis (Figure S6A) on days 10 and 17 following LPS exposure compared to PBS-treated samples, before declining slightly by day 28 (Figure 2A and B). This increase correlated with the presence of OCs within the woven bone, which peaked on days 10 and 17 and remained at intermediate levels on day 28, compared to the PBS group (Figure 2A). Although BMD declined on days 10 and 17, it returned to baseline by day 28 (Figure 2A and B and Figure S6B). Interestingly, by day 28, LPS-treated mice exhibited clear evidence of bone trabeculation (Figure S7). Further biomechanical analyses revealed that key parameters, including stiffness, yield load, maximum load, and work to fracture, were all elevated in LPS-treated mice compared to PBS-treated controls on day 28 (Figure 2C). Together, these findings suggest that periosteal bone formed in response to LPS undergoes remodeling and integrates with the existing bone, remains stable over time as the formation of new trabeculae is initiated. Ultimately, the overall bone structure is stronger relative to its pre-LPS state.

### LPS-induced periosteal bone formation positively correlates with bone marrow cell death

**Figure 3:**
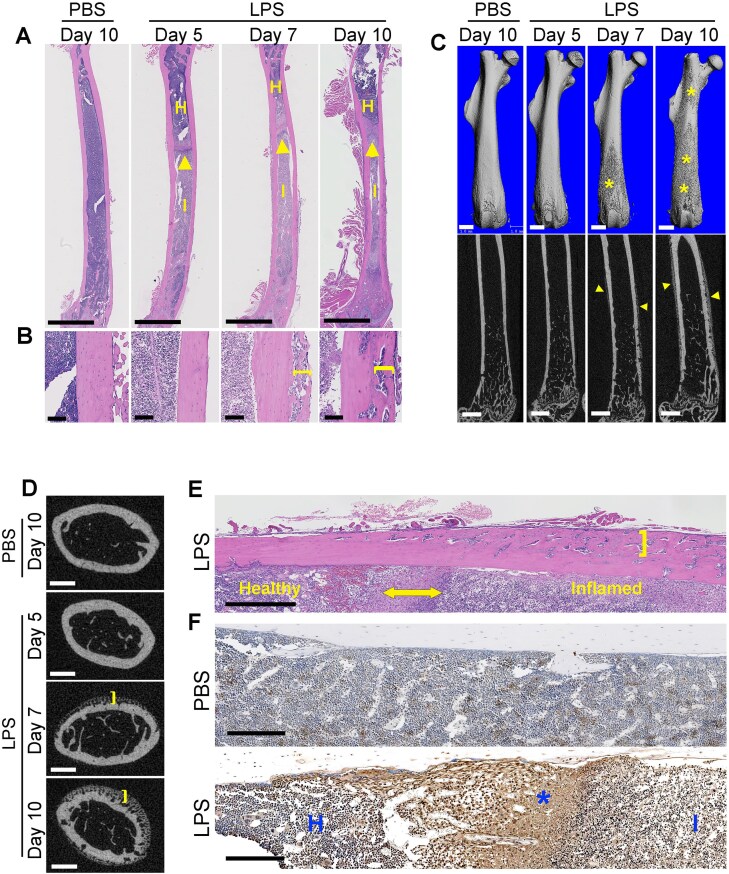
LPS-induced periosteal bone formation positively correlates with bone marrow cell death. Three-month-old male WT mice were i.p. injected with 1 mg/kg LPS or PBS (*n* = 3 for each group) on days 0, 4, and 8. Mice were sacrificed as indicated. Mice treated with PBS were sacrificed on day 10 as control (PBS). (A and B) Representative images of H&E stained-femoral sections. (A) Arrowheads show the interface between healthy (H) and inflamed (I) bone marrow. Scale bar: 2 mm. (B) Brackets indicate the thickness of the newly formed bone. Scale bar: 100 μm. (C) Representative μCT images of femoral diaphysis. Scale bar: 1 mm. Asterisks and arrowhead indicate areas of newly formed bone. (D) Representative μCT images of cross section of femora. Scale bar: 500 μm. Brackets indicate areas of newly formed bone. (E and F) Three-month-old WT mice were i.p. injected with 1 mg/kg LPS (*n* = 7) or PBS (*n* = 5) on days 0, 4, and 8. Mice were sacrificed on day 10. (E) Representative images of H&E-stained femoral sections. Double ended arrowhead shows the interface between healthy and inflamed pyroptotic bone marrow. Brackets indicate the thickness of the newly formed bone. Scale bar: 500 μm. (F) TUNEL staining. Staining is stronger in inflamed area (I) compared to heathy area (H) but weaker than in the interface zone (asterisk). Scale bar: 200 μm.

To understand the mechanisms by which LPS-induced periosteal bone formation, we analyzed samples from WT mice injected with LPS for different times. The color of bone marrow of LPS- and PBS-treated mice differed, most likely due to inflammation and cell death (Figure S8A and B). Notably, LPS primarily affected bone marrow cells, both CD45^+^ and CD45^−^ populations, whereas periosteal cells and splenocytes were largely unaffected (Figure S8B and C). The reason why CD45^+^ splenocyte populations were unaltered remains unclear. It also remains unknown whether discrete, localized cell death occurs in the periosteum or the adjacent muscle tissues, in addition the bone marrow. Nevertheless, bone marrow of LPS-exposed animals contained numerous leukocytes and displayed a layer of dead cells (arrowheads) interfacing healthy (H) and inflamed (I) areas (Figure 3A). While morphological changes in bone marrow were visible as early as 5 d after LPS injection, periosteal bone formation was not apparent until 7 d after LPS inoculation (Figure 3B). Micro-CT analysis confirmed that 7 d following LPS treatment, periosteal bone apposition was confined to the distal metaphysis but had extended to most of the diaphysis by day 10 (Figure 3C and D). Intriguingly, areas of early periosteal osteogenesis were prominently adjacent to those of compromised bone marrow compared to those of normal bone marrow (Figure 3E and Figure S8D). These observations were consistent with TUNEL staining, which revealed massive cell death in the interface zone compared to other marrow areas (Figure 3F and Figure S8E). Collectively, these results suggest that LPS induces bone marrow death, a response that precedes enhancement of cortical thickness.

### Noncanonical inflammasome mediates LPS-induced periosteal bone formation

**Figure 4:**
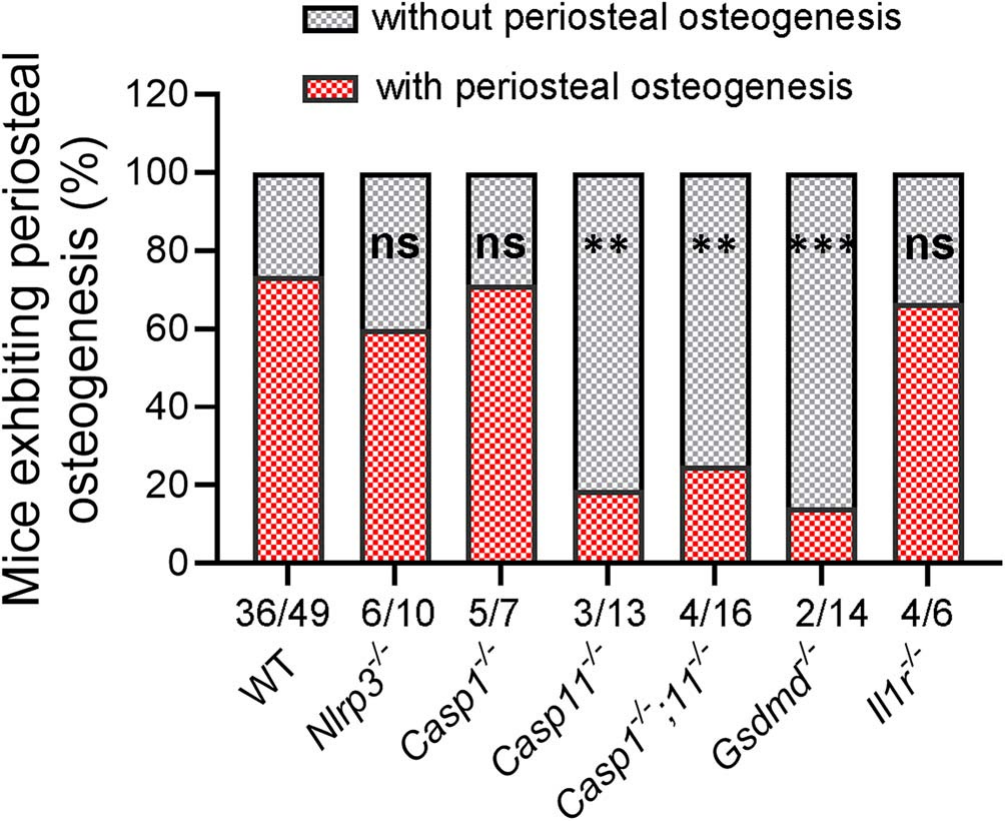
Non-canonical inflammasome mediates LPS-induced periosteal bone formation. Three-month-old male and female WT and mutant mice were i.p. injected with 1 mg/kg LPS from *E. coli* on days 0, 4, and 8. Mice were sacrificed on day 10 and femurs were collected for μCT analysis. The number of mice exhibiting periosteal bone formation/total number of mice tested is shown under the columns. Chi-Square test vs WT: ns, not significant. ^**^p < .01, ^***^p < .001.

To determine if LPS-induced bone marrow pyroptosis and understand the underlying mechanisms, we studied cortical bone outcomes in mice lacking key components of inflammasomes known to be regulated by LPS. These include NLRP3 canonical inflammasome (NLRP3, caspase-1), noncanonical inflammasome (caspase-11), and their shared substrate, GSDMD. Compared to WT mice, loss of NLRP3 or caspase-1 did not affect cortical bone formation (Figure 4). Lack of IL-1 receptor also failed to prevent LPS effects on bone in all treated mice (Figure 4 and Figure S9). By contrast, periosteal bone formation was significantly inhibited in mice deficient in *caspase-11*, both *caspase-1* and *caspase-11*, or *Gsdmd* (Figure 4). Accordingly, LPS-induced bone marrow hypocellularity in WT mice was prevented in *caspase-11^−/−^* and *Gsdmd^−/−^* counterparts (Figure S10A and B). Collectively, these data indicate that LPS primarily activates the noncanonical inflammasome-GSDMD axis to inflict bone marrow pyroptosis, a response that ultimately induces cortical bone formation.

### LPS-induced bone marrow pyroptosis releases osteogenic factors

**Figure 5:**
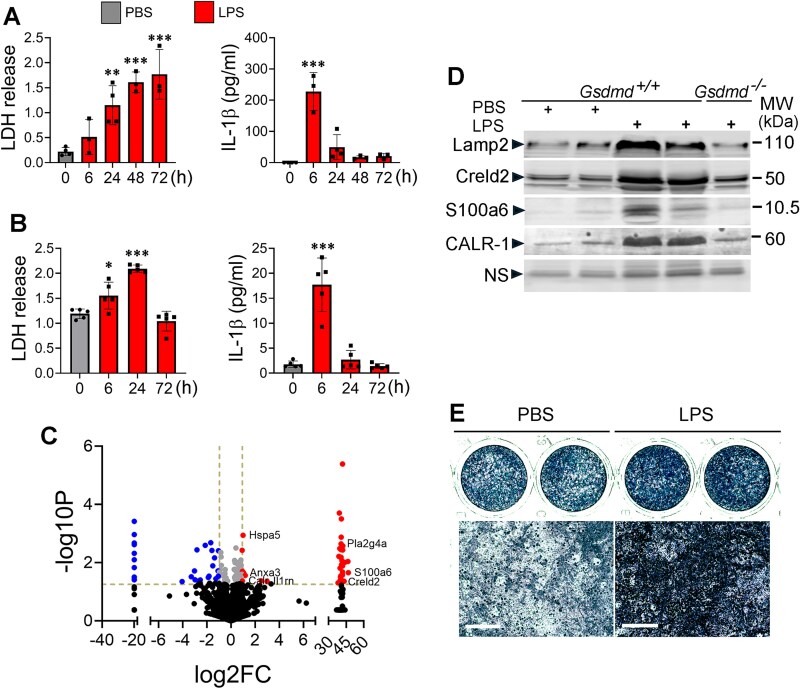
LPS-induced bone marrow pyroptosis releases osteogenic factors. (A and B) Three-month-old WT male mice were injected with PBS or LPS (*n* = 3-4) and sacrificed at the indicated time points. Bone marrow supernatants collected from the tibias and femurs centrifuged in tubes containing 100 μL PBS (A) and serum (B) were used for LDH (OD values) and IL-1β analysis. (C and D) Three-month-old WT mice were i.p. injected with PBS (*n* = 3) or 1 mg/kg LPS (*n* = 3) on day 0 and sacrificed on day 3. Bone marrow was centrifuged, and the supernatants were collected and analyzed by mass spectrometry. (C) Volcano plot. (D) Three-month-old WT and *Gsdmd^−/−^* mice were i.p. injected with PBS or LPS on day 0 and sacrificed on day 3. The supernatants from centrifuged bone marrow were analyzed by immunoblotting. Each lane represents a sample from an individual mouse. (E) Periosteal cells from WT mice were isolated with collagenase and expanded for 1 wk and cultured in osteogenic medium (50 μg/mL of ascorbic acid and 2 mM of glycerol 2-phosphate) for 3 d in the presence of bone marrow supernatants from mice treated with PBS or LPS for 3 d. Cells were stained for alkaline phosphatase activity. Top panels: images of whole wells. Bottom panels: high magnification images. Scale bar: 100 μm. Lamp2, lysosomal-associated membrane protein 2; Creld2, cysteine-rich with EGF-like domains 2; CALR, calreticulin; NS, nonspecific. ^*^p < .05, ^***^p < .001, ^****^p < .0001. One-way ANOVA.

To reinforce the conclusion that LPS caused bone marrow pyroptosis, we treated mice with PBS or 1 mg/kg LPS for various time points and monitored LDH and IL-1β levels in bone marrow supernatants. LPS induced a time-dependent release of LDH in bone marrow and blood, with significant differences observed at 24 and 6 h post-LPS injection, respectively, compared to PBS controls (Figure 5A and B). By contrast, LPS caused transient secretion of IL-1β as its levels were sharply increased 6 h after injection but returned to baseline values by 24 h.

GSDMD pores mediate the secretion of low molecular weight (MW) molecules (MW ≤18 kDa), such as IL-1β and IL-18.^42^^,^^43^ Therefore, we hypothesized that the presence in bone marrow fluid of larger cytoplasmic molecules (MW >18 kDa) in response to LPS should be the result of pyroptosis. To test this hypothesis, we employed mass spectrometry to analyze bone marrow supernatants from mice treated with PBS or LPS. Volcano plot showed proteins with increased or decreased levels upon LPS treatment (Figure 5C). Focusing on the proteins with increased levels, we found that most of them were cytoplasmic resident proteins. They included IL-1 receptor antagonist, Hspa5, calreticulin, S100a6, cPLA2α, Creld2, and annexin 3. To validate mass spectrometry results, we carried out immunoblotting analysis on bone marrow supernatants from mice treated with PBS or LPS, focusing on proteins for which reliable commercial antibodies are available. The levels of Lamp2, Creld2, S100a6, and CALR1 were higher in samples from LPS-exposed mice compared to PBS-treated controls, and this response was blunted in the absence of GSDMD (Figure 5D). To determine whether fluids containing this cocktail of molecules modulated osteogenesis following their release into the extracellular environment, we collected bone marrow supernatant from WT mice treated with LPS or PBS. Periosteal cells treated with LPS supernatants showed enhanced ALP activity and expression of *Alp* and *Col1a1* compared to those treated with PBS supernatants (Figure 5E and Figure S11). This effect was blunted when the supernatants were pretreated with proteinase K to digest and inactivate proteins or derived from GSDMD-deficient mice. Comparable results were obtained with osteoprogenitors isolated from bone marrow. Collectively, these findings suggest that the osteogenic activity of LPS supernatants is mediated by proteins released through GSDMD pores.

### Partial depletion of myeloid cells in bone marrow is sufficient to nearly blunt LPS-induced periosteal bone formation

**Figure 6:**
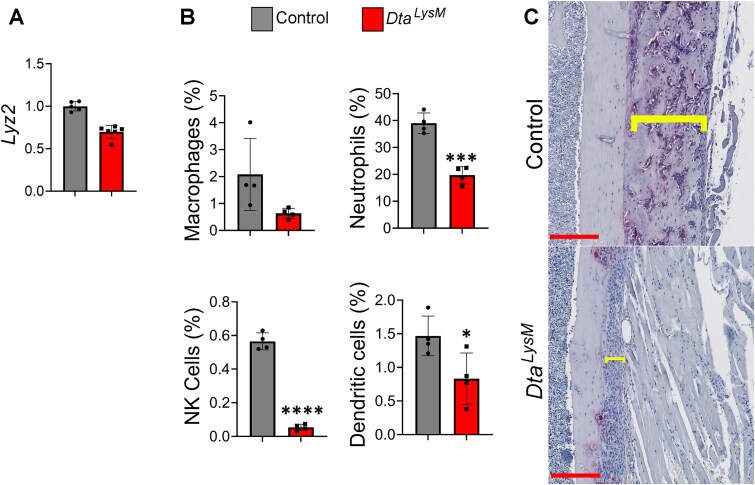
Partial depletion of myeloid cells in bone marrow is sufficient to blunt LPS-induced periosteal bone formation. Three-month-old WT and *Dta^LysM^* mice were used. Bone marrow cells were used for the analysis of *Lyz2* expression by qPCR (A) or cells by flow cytometry (B). Each black dot represents one mouse. Data are percentage of CD45^+^ cells (A and B). (C) Representative images of TRAP staining of femoral sections from WT (*n* = 5) or *Dta^LysM^* (*n* = 5) mice injected with 1 mg/kg LPS from *E. coli* on days 0, 4, and 8, sacrificed on day 10. Brackets indicate the thickness of the newly formed bone. Scale bar: 200 μm. ^*^p < .05, ^***^p < .001, ^****^p < .0001. Unpaired *t*-test.

The caspase-11-GSDMD axis is highly active in myeloid cells.^44^^,^^45^ To determine the impact of myeloid cell death on LPS-induced periosteal osteogenesis, we mated *Stop-floxed diphtheria toxin A* (*DtA*) mice with *lysozyme M* (*LysM*)*-Cre* mice to generate *Stop-Dta*;*LysM-Cre* (*DtA^LysM^*) and control mice. The analysis of the expression LysM (encoded by *lyz2*) in bone marrow by qPCR (Figure 6A) and FACS (Figure 6B and Figure S12A and B) confirmed partial ablation of bone marrow myeloid cells. LPS-induced periosteal bone formation was significantly reduced in *DtA^LysM^* compared with WT control (Figure 6C). Thus, LPS-induced death of myeloid cells is involved in periosteal bone outcomes.

### LepR^+^ periosteal osteoprogenitors contribute to LPS-induced periosteal bone formation

**Figure 7:**
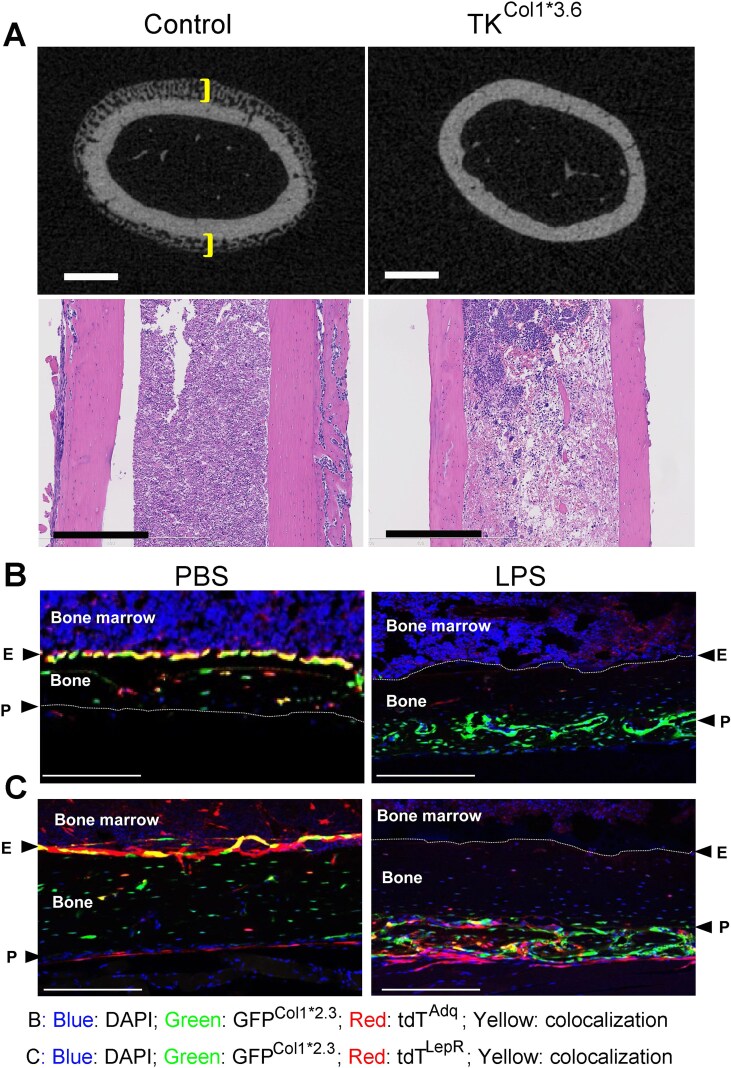
Newly formed periosteal bone induced by LPS is derived from LepR^+^ periosteal SSCs. (A) Three-month-old male WT (control; *n* = 11) and Tk^Col1^*^3.6^ mice (*n* = 10) were i.p. injected with 1 mg/kg LPS on days 0, 4, and 8. All mice were i.p. injected with 8 μg/g ganciclovir (GCV), twice daily from day 0 to day 9. Femurs were collected on day 10 and analyzed by μCT and H&E staining. Representative images of femoral diaphysis. Brackets indicate the thickness of the newly formed bone. Scale: upper panel: 500 μm. Lower panels: H&E staining; scale bar: 400 μm. (B and C) Femurs from 3-mo-old male *tdT^Adq^*; GFP^Col1^*^2.3^ mice (B) and *tdT^LepR^*; GFP^Col1^*^2.3^ (C) mice were i.p. injected with PBS (left panel; *n* = 3) or 1 mg/kg LPS (right panel; *n* = 3) on days 0, 4, and 8 and analyzed on day 10. Representative images are shown. Blue, DAPI; red, tdT^Adq^ (B) or tdT^LepR^ (C); green, GFP; E, endosteum; P, periosteum. Scale bar: 200 μm.

Bone comprises inner endosteal and outer periosteal compartments with common and likely unique cell populations.^8^ Our results showing profound periosteal bone formation in LPS-stimulated mice raise the possibility that osteoprogenitors in the periosteum, but not in bone marrow are substantially increased. It has been reported that transgenes driven by 3.6 kb Col1a1 promoter (col1^*^3.6) target both endosteal and periosteal immature OBs.^46^ To determine if periosteal and bone marrow osteoprogenitors are impacted by LPS, we generated mice in which col1^*^3.6 drove the expression of TK (Tk^Col1^*^3.6^) to which we administered ganciclovir to deplete osteoprogenitors.^38^^,^^39^ We found that while depletion of Tk^Col1^*^3.6^ cells upon ganciclovir administration did not affect LPS-induced bone marrow cell pyroptosis, periosteal bone formation was eliminated in these mice (Figure 7A), indicating that cortical bone was formed by periosteal osteoprogenitors.

It has been reported that Adq^+^ cells and LepR^+^ cells differentiate into OBs both in vitro and in vivo. To assess the contribution of Adq^+^ and LepR^+^ cells to LPS outcomes, we crossed *stop*-*floxed-tdTomato* (*tdT^fl/+^*), *2.3Col1GFP* (GFP^Col1^*^2.3^), with either *Adq-Cre* (*Adq^Cre^*) or *Lepr-Cre* (*Lepr^Cre^*) mice to respectively generate Adq^Cre^; tdT; GFP^Col1^*^2.3^ (*tdT^Adq^*) mice and Lepr^Cre^; tdT; GFP^Col1^*^2.3^ (*tdT^LepR^*) mice and monitored the colocalization of labeled cells. 2.3Col1 was used as a marker of mature OBs.^46^^,^^47^ In PBS treated-mice, while *tdT^Adq^* only labels endosteum (Figure 7B, left  panel), *tdT^lepR^* targets both endosteum and periosteum (Figure 7C, left  panel). After LPS stimulation, *tdT^Adq^* did not target any GFP^Col1^*^2.3+^ OBs in the newly formed periosteal bone (Figure 7B, right  panel), but *tdT^LepR^* targeted approximately 15% GFP ^Col1^*^2.3+^ OBs in this new tissue (Figure 7C, right  panel; Figure S13). This suggests that the newly formed periosteal OBs are not derived from Adq^+^ cells, but LepR^+^ cells contribute, at least in part, to periosteal bone formation following LPS stimulation.

## Discussion

LPS primes canonical inflammasomes, such as those formed by NLRP3, and directly triggers noncanonical inflammasomes, resulting in the release of IL-1β and IL-18 or induction of pyroptosis.^28–30^ In this study, we unexpectedly found that low systemic levels of LPS not only caused inflammation and bone resorption as expected but also led to significant periosteal bone accrual, which underwent remodeling and integrates with the existing bone, resulting in a stronger overall bone structure compared to its pre-LPS state. Using various genetic mouse models, we showed that this periosteal response was associated with pyroptosis of both CD45^+^ cells and CD45^−^ cells in the bone marrow driven by the caspase-11-GSDMD pathway. The exact reason why the LPS regimen used in this study induced pyroptosis and cortical bone response remains unclear. However, a recent meta-analysis highlighted that the effects of LPS on trabecular and cortical bone structures varied depending on the dose, route of administration, and duration of exposure.^34^ Given the complexity of LPS’s effects, further research is needed to better understand the mechanisms driving the responses observed in this study.

The release of intracellular contents into the extracellular space is a critical step in the inflammatory responses induced by lytic cell death. In addition to IL-1β, IL-18, and various other proteins, pyroptotic cells also released metabolites and eicosanoids like PGE_2_, which were shown to promote wound healing.^48^^,^^49^ Moreover, remnants of pyroptotic cells, often crowned with F-actin-rich filopodia, have been suggested as a mechanism for initiating adaptive immune responses.^49^ Our findings revealed that LPS-induced pyroptosis in bone marrow cells led to the release of several proteins, most of which were resident intracellular macromolecules. We confirmed that bone marrow supernatants from *Gsdmd^+/+^* mice, but not *Gsdmd^−/−^* littermates treated with LPS, enhanced OB differentiation in vitro when compared to supernatants from PBS-treated mice. Collectively, these findings highlight a novel mechanism in which pyroptotic cells release molecules that directly or indirectly promote the differentiation of neighboring mesenchymal cells. Thus, in addition to its established roles in limiting replicative niches during infection, propagating inflammation, promoting tissue repair, and triggering adaptive immune responses,^48^^,^^49^ pyroptosis also plays a pivotal role in facilitating cell differentiation.

Osteogenesis is initiated by the proliferation and differentiation of mesenchymal stem cells. While the mesenchymal nature of these cells is well established, their precise identification remains challenging. Bone marrow Adq^+^ cells have the potential to differentiate into OBs, and within adult bone marrow, LepR^+^ osteoprogenitors are the primary source of new OBs for bone maintenance. Although Adq^+^ and LepR^+^ cells almost entirely overlap in bone marrow, periosteal cells are predominantly targeted by LepR^+^ cells, rather than Adq^+^ cells. Our findings suggest that bone formation occurred in the periosteum but not in the endosteum, likely because osteoprogenitors survived in the periosteal envelope but were rapidly lost in the bone cavity marrow following LPS exposure. As pyroptosis-driven inflammation gradually resolves, trabecular bone formation is subsequently initiated. Lineage tracing data further confirmed that tdT^LepR^ cells, but not tdT^Adq^ cells, targeted OBs in the newly formed periosteum. These results suggest that: (1) newly differentiated periosteal OBs did not originate from Adq^+^ cells, and LepR^+^ cells were at least partially responsible for the periosteal bone formation induced by LPS; (2) bone marrow Adq^+^ cells were unable to migrate into the periosteum, a view that is consistent with the massive cell death in the bone marrow compartment.

LPS induced the death of CD45^−^ cells in the bone marrow, but not in the periosteum. Similarly, it led to the loss of tdT^+^LepR^+^ cells in the bone marrow while sparing those in the periosteum. In addition, osteoprogenitors from both compartments exhibited enhanced in vitro osteogenic potential when exposed to supernatants from LPS-treated mice. Together, these findings support our working model that LPS promotes bone formation in the periosteum, but not in the endosteum, likely due to compartment-specific differences in osteoprogenitor survival. However, several critical questions remain to be addressed to fully support this model. First, do macromolecules released by pyroptotic cells influence bystander cells extracellularly, or is internalization required? Precedent for extracellular activity of intracellular macromolecules exists; for example, histones and DNA can propagate inflammation when released, as seen with macrophage and neutrophil extracellular traps. Second, how do factors released from pyroptotic bone marrow cells traverse the cortical bone to reach periosteal osteoprogenitors? In this context, might these molecules be transported via transcortical blood vessels,^29^^,^^30^^,^^50^ diffuse along the vascular channels and/or act on osteocytes near the endosteum, initiating signaling through the lacunar-canalicular networks^31^^,^^32^ to promote periosteal bone formation? Despite these unresolved questions, our study underscores the concept that pyroptosis is not merely an endpoint, but rather a dynamic response that triggers downstream cascades. Indeed, we report that the occurrence of pyroptosis in bone marrow myeloid cells promotes not only the expected inflammation-associated osteoclastogenesis but also osteogenesis.

## Supplementary Material

Supplumentary_Figures_zjaf197

ARRIVE-_Animals_in_Research_Reporting_In_Vivo_Experiments_zjaf197

## Data Availability

All data supporting the findings of this study are included in the article and its supplementary materials. Raw imaging, histology, and other experimental data are available from the corresponding author upon reasonable request.
